# Optimization of acute medication use following eptinezumab initiation during a migraine attack: post hoc analysis of the RELIEF study

**DOI:** 10.1186/s10194-022-01463-3

**Published:** 2022-07-28

**Authors:** Roger Cady, Richard B. Lipton, Dawn C. Buse, Mette Krog Josiassen, Annika Lindsten, Anders Ettrup

**Affiliations:** 1 RK Consults, Ozark, MO United States; 2 Missouri State University, Springfield, MO United States; 3 Lundbeck LLC, Deerfield, IL United States; 4 Department of Neurology, Albert Einstein College of Medicine, Bronx, NY United States; 5 Vector Psychometric Group, LLC, Chapel Hill, NC United States; 6 H. Lundbeck A/S, Copenhagen, Denmark

**Keywords:** Migraine, Eptinezumab, CGRP, mTOQ

## Abstract

**Background:**

The benefits of preventive treatment on the effectiveness of migraine management have rarely been examined. This post hoc analysis investigated the impact of eptinezumab on the optimization of acute medication effectiveness using the 4-item Migraine Treatment Optimization Questionnaire (mTOQ-4) to measure acute medication optimization over 4 weeks post-infusion.

**Methods:**

RELIEF was a 12-week, phase 3, multicenter, parallel-group, double-blind, placebo-controlled clinical trial conducted in patients aged 18–75 years with a ≥ 1-year history of migraine and 4–15 migraine days per month in the 3 months prior to screening. Patients were randomized 1:1 to a 30-min infusion of eptinezumab 100 mg or placebo within 1–6 h of a qualifying migraine attack. The mTOQ-6 and 6-item Headache Impact Test (HIT-6) were administered at screening visit and week 4. From the mTOQ-6, we calculated the mTOQ-4 using the following items: “2-h pain free,” “24-h relief,” “able to plan,” and “feeling in control” to measure acute medication optimization.

**Results:**

A total of 238 patients received eptinezumab 100 mg and 226 provided week 4 data; 242 received placebo and 232 provided week 4 data. In the eptinezumab arm, the proportion of patients with moderate/maximal optimization increased from 31.4% at baseline to 58.0% (26.6 percentage point increase) at week 4. The corresponding proportions in the placebo group were 40.5% to 50.4% (9.9 percentage point increase). Eptinezumab treatment was associated with numerically larger improvements in HIT-6 at week 4. Relative improvements with eptinezumab vs. placebo from baseline to week 4 in HIT-6 were greater in those with poor treatment optimization at baseline.

**Conclusions:**

In comparison with placebo, treatment with eptinezumab was associated with improvements in acute medication optimization as measured by mTOQ and reductions in headache impact, as measured by HIT-6. These benefits were greater in those with poor acute treatment optimization prior to preventive treatment with eptinezumab.

**Trial registration:**

ClinicalTrials.gov identifier: NCT04152083.

## Introduction

Migraine is among the world’s most disabling disorders, ranking second globally for years of life lived with a disability [1]. The estimated prevalence of migraine is 14% globally [2]. Despite the high burden of migraine, highlighting the need for effective therapy, migraine remains underdiagnosed and undertreated [3]. Less than half of those with migraine seek medical care and receive a migraine diagnosis [4]. Ideally, preventive treatment should reduce the frequency, severity, and duration of migraine episodes, while slowing disease progression [5]. Acute treatment should safely and rapidly alleviate an episode, within 2 h of treatment [6]. Yet, effective acute treatment of migraine episodes remains a challenge; approximately 34% of patients respond poorly to treatment, which may lead to increased severity in symptoms and disability levels [7]. If the acute medication is not working optimally, it renders the patient at risk for a vicious cycle that can lead to migraine disease progression and medication-overuse headache (MOH) [8]. Furthermore, ineffective acute treatment in patients with episodic migraine is associated with an increased risk of chronic migraine onset, sometimes referred to as migraine chronification [9, 10]. The majority of individuals with migraine use acute therapies, including both over-the-counter and prescription options [9]. Despite advances in preventive treatment, including the development of the calcitonin gene-related peptide (CGRP)-targeted monoclonal antibodies and gepants, acute medications continue to be needed by most patients to manage breakthrough attacks of migraine [11].

Treatment with eptinezumab, a humanized IgG1 monoclonal antibody that binds the CGRP ligand with high affinity, was associated with statistically significant reductions in mean monthly migraine days over weeks 1–12 in comparison with placebo for both episodic and chronic migraine (PROMISE-1 and PROMISE-2) [12–14]. Another phase 3, multicenter, double-blind trial (RELIEF) in patients eligible for preventive migraine treatment (experiencing migraine 4–15 days/month), found that eptinezumab, administered during a migraine attack, was associated with reduction in headache pain intensity and most bothersome migraine-associated symptoms. Eptinezumab also resulted in less use of rescue medication during the attack as compared with placebo [15]. Additionally, when compared to placebo, eptinezumab-treated patients demonstrated greater improvement in acute treatment response during subsequent migraine attacks as measured by the prespecified 6-item Migraine Treatment Optimization Questionnaire (mTOQ-6) total score [16].

Previous research showed that mTOQ-4, a short-form of mTOQ-6, was a reliable tool for assessing acute medication optimization [9]. This post hoc analysis of the RELIEF study was conducted to investigate the impact of eptinezumab on the optimization of acute medication use, using mTOQ-4, over 4 weeks following administration during a migraine attack in patients eligible for preventive treatment. Secondary analyses included evaluating changes in headache-related impact and acute medication optimization across patients grouped by their baseline acute medication optimization.

## Methods

### Study design and patients

Detailed methodology for RELIEF has been published [15]. Briefly, RELIEF was a 4- to 12-week, phase 3, multicenter, parallel-group, double-blind, placebo-controlled clinical trial conducted between November 2019 and July 2020 (NCT04152083) in which patients were randomized to receive eptinezumab 100 mg or placebo. Patients were between the ages of 18–75 years (inclusive) with a ≥ 1-year history of migraine (defined by the International Classification of Headache Disorders, 3^rd^ edition [ICHD-3] criteria [5]), with or without aura, with onset of first migraine before age 50 years, and migraine on 4–15 days per month in the 3 months prior to screening. Patients were required to have typical migraine attacks with a duration of 4–72 h if untreated, with headache pain of moderate to severe intensity and a prespecified most bothersome symptom of nausea, photophobia, or phonophobia. Additionally, patients were required to have a history of either previous or active use of triptans for acute treatment of migraine. Treatment (total volume of 100 mL) was administered intravenously over a period of 30 min on day 0 within 1–6 h of onset of the qualifying migraine attack of moderate to severe intensity.

### Outcome measures

Patient-reported outcome measures included the mTOQ-6 and 6-item Headache Impact Test (HIT-6). The mTOQ-6 and HIT-6 were captured at baseline and at week 4. The mTOQ-6 data was transformed into the mTOQ-4 for the purposes of these analyses.

The mTOQ-6 is a self-report questionnaire used to assess the optimization of acute treatment in persons with migraine. The 6 mTOQ items are as follows: (1) “Are you able to quickly return to your normal activities (i.e., work, family, leisure, social activities) after taking your migraine medication?” (2) “Can you count on your migraine medication to relieve your pain within 2 h for most attacks?” (3) Does one dose of your migraine medication usually relieve your headache and keep it away for at least 24 h?” (4) “Is your migraine medication well tolerated?” (5) “Are you comfortable enough with your migraine medication to be able to plan your daily activities?” (6) “After taking your migraine medication, do you feel in control of your migraines enough so that you feel there will be no disruption to your daily activities?” [17]. Each of the 6 items is scored as never (1), rarely (2), less than half the time (3), and half the time or more (4). The mTOQ-6 total score is calculated by summing individual question scores (score range of 6‒24), with higher scores indicating better acute treatment optimization [17].

In addition to the mTOQ-6 scores, the developers provide a simplified scoring for the mTOQ-4 by selecting the items that best assessed treatment efficacy: “2-h pain free,” “24-h relief,” “able to plan,” and “feeling in control” [9]. Items evaluating “tolerability” and “quick return to function” were excluded because they were either unrelated or redundant [9]. For analysis purposes, the item scores were classified (combining rarely and less than half the time) and new scores were assigned as never (0), rarely (0), less than half the time (1), and at least half the time (2), and then summed for total score. Patients were then grouped by baseline mTOQ-4 total scores into the following optimization categories: very poor (0), poor (1–5), moderate (6–7), and maximal (8). A simplified grouping combined very poor with poor (0–5) and moderate with maximal (6–8).

The HIT-6 is a self-reported assessment of the impact on the ability to function normally in daily life when a headache occurs [18]. Each of the 6 items is scored as never (6), rarely (8), sometimes (10), very often (11), and always (13). The HIT-6 total score is calculated by summing individual items (score range of 36‒78 points), with the total score representing headache-related life impact: severe impact =  ≥ 60, substantial impact = 56–59, some impact = 50–55, and little to no impact =  ≤ 49.

### Statistical analysis

For the mTOQ-6 and HIT-6, total scores were summarized by treatment group at baseline and week 4 (or early termination) and change from baseline to the week 4 visit was calculated. If response was missing for ≥ 1 item of the HIT-6 or mTOQ-6, the respective total score was treated as missing; no missing data were imputed. As a post hoc analysis, all results are summarized descriptively.

## Results

Baseline demographics and characteristics have been reported and showed similarity between eptinezumab and placebo groups [15]. A total of 238 and 242 patients were randomized to receive eptinezumab 100 mg or placebo, respectively; week 4 data were available for the present analysis for 226 patients treated with eptinezumab and 232 receiving placebo.

**Fig. 1:**
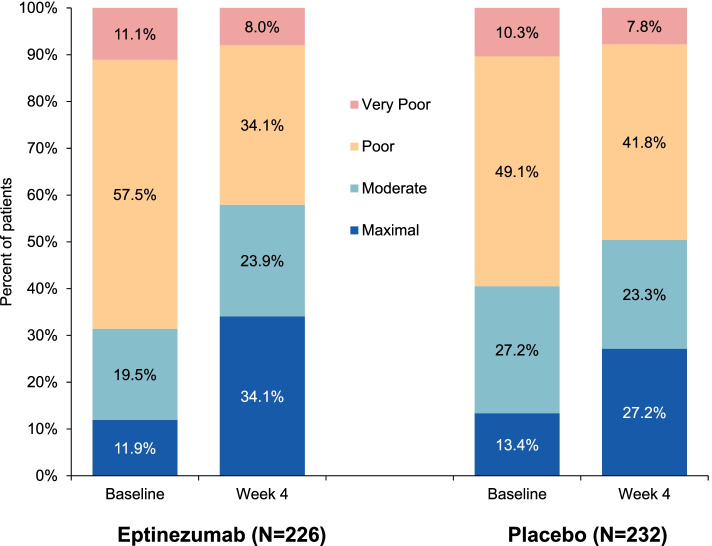
Distribution of acute medication optimization (mTOQ-4) at baseline and week 4. The mTOQ-4 was derived from the mTOQ-6 by selecting the items that best assessed efficacy: “2-h pain free,” “24-h relief,” “able to plan,” and “in control.” Each item is rated never (1), rarely (2), less than half the time (3), or half the time or more (4). Patients were grouped by baseline mTOQ-4 total scores into the following optimization categories: very poor (0), poor (1–5), moderate (6–7), and maximal (8). mTOQ-4/mTOQ-6, 4-item/6-item Migraine Treatment Optimization Questionnaire

**Fig. 2:**
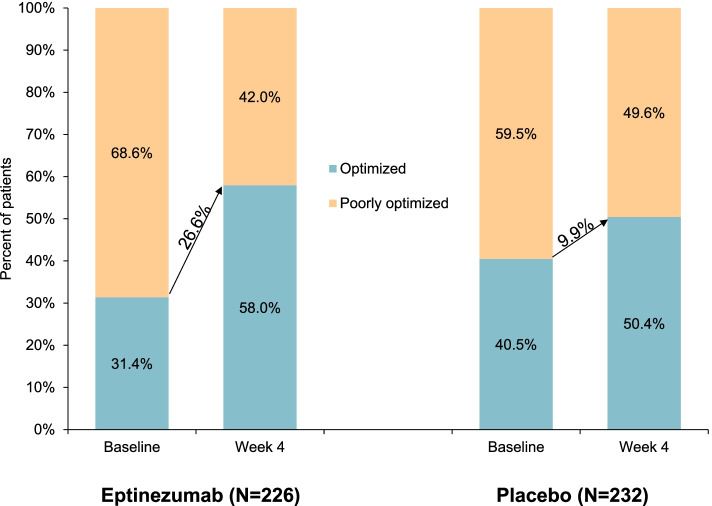
Simplified distribution of acute medication optimization (mTOQ-4) at baseline and week 4. The mTOQ-4 was derived from the mTOQ-6 by selecting the items that best assessed efficacy: “2-h pain free,” “24-h relief,” “able to plan,” and “in control.” Each item is rated never (1), rarely (2), less than half the time (3), or half the time or more (4). Poorly optimized comprises the “very poor” and “poor” categories; optimized comprises “moderate” and “maximal.” Simplified subgroups consist of patients with mTOQ-4 total scores in very poor (0) and poor (1–5) categories together, along with patients with total scores in the moderate (6–7) and maximal (8) categories grouped together. mTOQ-4/mTOQ-6, 4-item/6-item Migraine Treatment Optimization Questionnaire

At baseline, the greatest proportion of patients in both groups had poor acute treatment optimization, with less than 14% having maximal optimization (Fig. 1). The distribution at post-treatment week 4 shows a greater shift toward maximal acute treatment optimization, with an increase over baseline of 22.2 percentage points for the eptinezumab treatment group compared with 13.8 percentage points for the placebo group (Fig. 1). Acute treatment optimization distributions were simplified into “poorly optimized” (“very poor” and “poor” categories) and “optimized” (“moderate” and “maximal” categories). The percentage of patients who were “optimized” increased by 26.6 percentage points with eptinezumab compared with 9.9 percentage points with placebo (Fig. 2). Of the 155 eptinezumab-treated patients who were very poorly or poorly optimized at baseline, 73 (47.1%) were moderately or maximally optimized at week 4, respectively; in comparison, of the 138 patients receiving placebo, 35 (25.4%) were moderately or maximally optimized.

**Fig. 3:**
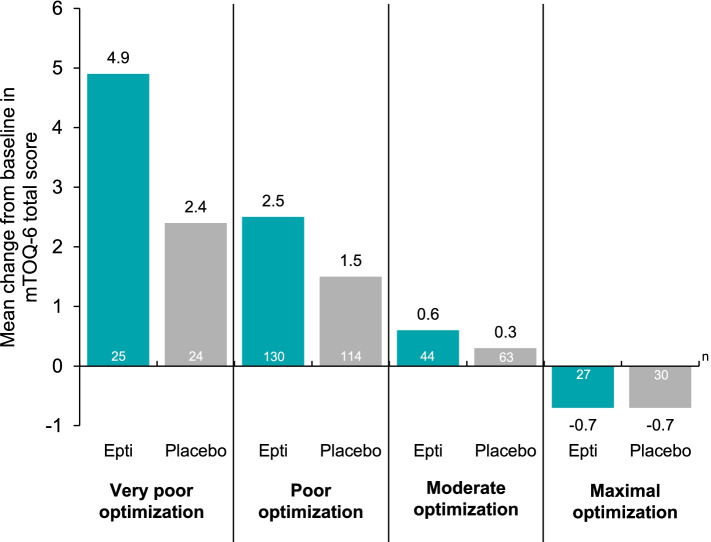
Change from baseline to week 4 in mTOQ-6 total score by baseline acute treatment optimization subgroup. Patients were grouped by baseline mTOQ-4 total scores into the following optimization categories: very poor (0), poor (1–5), moderate (6–7), and maximal (8). The mTOQ-6 total score ranges from 6 to 24, with higher scores indicating better acute medication optimization. Epti, eptinezumab (100 mg); mTOQ-4/mTOQ-6, 4-item/6-item Migraine Treatment Optimization Questionnaire

Mean mTOQ-6 total scores at baseline and week 4 by baseline acute treatment optimization subgroups are summarized in Table 1. Across subgroups, eptinezumab treatment resulted in numerically larger improvements than placebo in mTOQ-6 total score at week 4, with a trend of larger improvements in subgroups with worse optimization at baseline (Fig. 3). Mean change from baseline to week 4 in mTOQ-6 total score in eptinezumab-treated patients very poorly optimized at baseline was 4.9 versus 2.4 in patients receiving placebo (Fig. 3).

**Table 1:** mTOQ-4 total score at baseline and week 4 by baseline acute treatment optimization subgroup

		**Eptinezumab 100 mg**	**Placebo**
**Optimization subgroup**	**Time point**	**Mean (SD) [n]**	**Mean (SD) [n]**
Very poor	Baseline	11.2 (2.4) [25]	11.6 (2.2) [25]
	Week 4	16.2 (5.3) [25]	13.9 (5.5) [24]
	Change from baseline	4.9 (4.9) [25]	2.4 (5.1) [24]
Poor	Baseline	17.0 (2.6) [138]	16.8 (2.7) [117]
	Week 4	19.6 (4.1) [130]	18.4 (3.3) [114]
	Change from baseline	2.5 (4.2) [130]	1.5 (3.0) [114]
Moderate	Baseline	21.5 (1.2) [46]	21.9 (1.0) [65]
	Week 4	22.1 (2.1) [44]	22.2 (2.0) [63]
	Change from baseline	0.6 (2.0) [44]	0.3 (1.8) [63]
Maximal	Baseline	23.8 (0.5) [28]	23.8 (0.5) [35]
	Week 4	23.1 (1.8) [26]	23.2 (2.0) [30]
	Change from baseline	-0.7 (1.9) [26]	-0.7 (2.0) [30]
**Simplified subgroups**			
Poorly optimized	Baseline	16.1 (3.3) [163]	15.9 (3.3) [142]
	Week 4	19.0 (4.5) [155]	17.6 (4.1) [138]
	Change from baseline	2.9 (4.4) [155]	1.7 (3.5) [138]
Optimized	Baseline	22.4 (1.5) [75]	22.6 (1.2) [100]
	Week 4	22.5 (2.0) [71]	22.6 (2.0) [93]
	Change from baseline	0.1 (2.1) [71]	0.0 (1.9) [93]

Patients were grouped by baseline mTOQ-4 total scores into the following optimization categories: very poor (0), poor (1–5), moderate (6–7), and maximal (8). Simplified subgroups consist of patients with mTOQ-4 total scores in very poor (0) and poor (1–5) categories together, along with patients with total scores in the moderate (6–7) and maximal (8) categories grouped together. Epti, eptinezumab (100 mg); mTOQ-4/mTOQ-6, 4-item/6-item Migraine Treatment Optimization Questionnaire

**Fig. 4:**
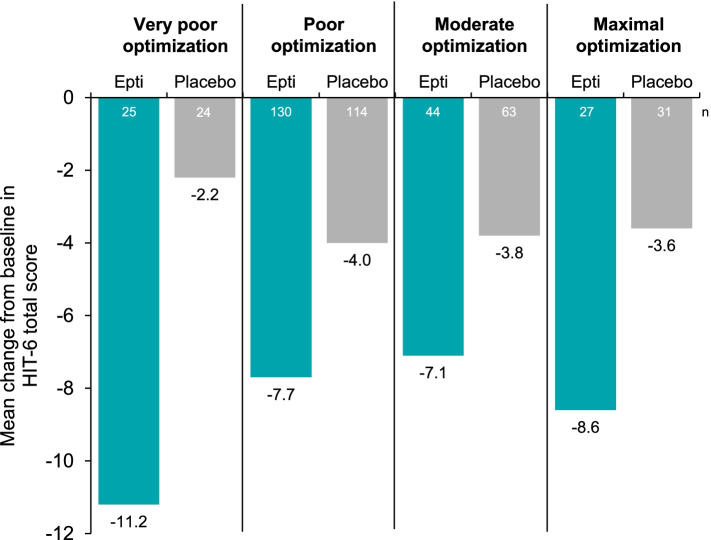
Change from baseline to week 4 in HIT-6 total score by baseline acute treatment optimization subgroup based on mTOQ-4. Patients were grouped by baseline mTOQ-4 total scores into the following optimization categories: very poor (0), poor (1–5), moderate (6–7), and maximal (8). The HIT-6 total score ranges from 36 to 78, with higher scores indicating more severe headache-related impact. Epti, eptinezumab (100 mg); HIT-6, 6-item Headache Impact Test; mTOQ-4, 4-item Migraine Treatment Optimization Questionnaire

Mean HIT-6 total scores at baseline were 66.6–69.6, 65.0–66.0, 63.2–64.7, and 62.5–63.5 points in the very poor, poor, moderate, and maximal optimization subgroups, respectively (Table 2). Similar to the mTOQ-6, eptinezumab treatment was associated with numerically larger improvements than placebo in HIT-6 total score at week 4 (Fig. 4). Patients with very poor baseline optimization reported the greatest amount of change (‒11.2 with eptinezumab vs. ‒2.2 with placebo from baseline to week 4) (Fig. 4).

**Table 2:** HIT-6 total score at baseline and week 4 by baseline acute treatment optimization subgroup

		**Eptinezumab 100 mg**	**Placebo**
**Optimization subgroup**	**Time point**	**Mean (SD) [n]**	**Mean (SD) [n]**
Very poor	Baseline	69.6 (4.6) [25]	66.6 (4.6) [25]
	Week 4	58.4 (11.8) [25]	64.5 (7.5) [24]
	Change from baseline	-11.2 (12.2) [25]	-2.2 (6.9) [24]
Poor	Baseline	65.0 (4.6) [138]	66.0 (4.6) [117]
	Week 4	57.1 (9.5) [130]	62.0 (7.8) [114]
	Change from baseline	-7.7 (10.3) [130]	-4.0 (7.2) [114]
Moderate	Baseline	64.7 (4.6) [46]	63.2 (5.2) [65]
	Week 4	57.3 (8.9) [44]	59.4 (7.6) [63]
	Change from baseline	-7.1 (9.0) [44]	-3.8 (8.5) [63]
Maximal	Baseline	63.5 (5.2) [29]	62.5 (4.8) [35]
	Week 4	54.7 (10.1) [27]	58.7 (7.8) [31]
	Change from baseline	-8.6 (11.0) [27]	-3.6 (8.4) [31]
**Simplified subgroups**			
Poorly optimized	Baseline	65.7 (4.9) [163]	66.1 (4.6) [142]
	Week 4	57.3 (9.9) [155]	62.4 (7.7) [138]
	Change from baseline	-8.3 (10.7) [155]	-3.7 (7.2) [138]
Optimized	Baseline	64.3 (4.8) [75]	62.9 (5.0) [100]
	Week 4	56.4 (9.4) [71]	59.2 (7.6) [94]
	Change from baseline	-7.7 (9.7) [71]	-3.7 (8.5) [94]

Patients were grouped by baseline mTOQ-4 total scores into the following optimization categories: very poor (0), poor (1–5), moderate (6–7), and maximal (8). The HIT-6 total score ranges from 36 to 78, with higher scores indicating more severe headache-related impact. Simplified subgroups consist of patients with mTOQ-4 total scores in very poor (0) and poor (1–5) categories together, along with patients with total scores in the moderate (6–7) and maximal (8) categories grouped together. Epti, eptinezumab (100 mg); HIT-6, 6-item Headache Impact Test; mTOQ-4, 4-item Migraine Treatment Optimization Questionnaire

## Discussion

Primary results from the RELIEF study showed that eptinezumab, when administered within 1‒6 h of a migraine attack, provided shorter time to headache pain freedom and absence of most bothersome symptom after the start of infusion compared with placebo [15]. In this post hoc analysis, the effects of eptinezumab compared with placebo on acute treatment optimization and headache-related impact were explored in patients grouped by their level of acute medication optimization at baseline. These analyses from the RELIEF study show that eptinezumab, after demonstrating effects on the active migraine attack, resulted in numerically larger improvements in the proportion of patients with optimized acute medication at week 4 and was associated with numerically larger improvements in mTOQ-6 total score and HIT-6 total score at week 4 when compared with placebo. Given its half-life of 27 days [19], eptinezumab may have a unique role in effectively treating an active migraine attack and remaining active to work synergistically with traditional acute treatments to optimize treatment of future breakthrough migraine attacks.

The mTOQ-6 was transformed into the mTOQ-4 in alignment with the developers’ modification, which has demonstrated usefulness as a tool in determining acute medication optimization and thus is useful in determining how preventive treatment may impact the effectiveness of acute medication [9]. Results from this post hoc analysis showed that, numerically, more patients treated with eptinezumab shifted from having poorly optimized to optimized acute treatment. In addition, the trend of larger improvements in mTOQ-6 total score in patients with poorer optimization at baseline suggests that eptinezumab may work synergistically with acute medications to better control acute breakthrough attacks of migraine. Likewise, better response to acute intervention may work synergistically with preventive treatment to prevent migraine chronification.

Changes from baseline in HIT-6 total score did not show a clear trend of increasing improvement with poorer baseline optimization; however, eptinezumab treatment was associated with numerically larger improvements than placebo in HIT-6 total score at week 4, similar to the mTOQ-6 [16]. The most pronounced effects were noted in very poorly optimized patients, which may include patients who are potentially stuck in the vicious cycle of medication overuse and MOH. These data highlight the potential benefit of eptinezumab in optimizing acute medication use during breakthrough attacks and suggest that eptinezumab may reduce the overall impact and severity of migraine, potentially lowering the risk of migraine chronification.

There is currently a lack of studies that demonstrate a causative link between effective preventive migraine treatment and improved acute medication optimization. Previous research has indicated that acute medication is less effective in patients with higher frequency of monthly headache days, more severe migraine attacks, and MOH, with current use of preventive migraine treatment associated with increased effectiveness [20]. The recent introduction of CGRP-targeting therapy in clinical practice presents the opportunity to find appropriate conditioning, and potentially combination therapy, of different drug classes (antidepressants, triptans, gepants, onabotulinumtoxinA) [21]. Though a causal effect cannot be determined in the current post hoc analysis, the results suggest that eptinezumab—which has been shown to reduce migraine and headache frequency and severity and days of acute headache medication use in patients with episodic migraine [13, 22], chronic migraine [23–25], and chronic migraine with MOH [26, 27]—may prevent chronification, which could aid in decoupling the cycle of chronic migraine and MOH [8], and that eptinezumab may have an enduring effect on acute treatment optimization.

### Limitations

These analyses are primarily limited by their post hoc nature, where only summary statistics were reported, and by smaller sample sizes of some subgroups. To date, there are no other reports that have studied mTOQ-4 scores in patients before and after initiation of preventive migraine treatment. However, the design of RELIEF limits the ability to connect the preventive migraine efficacy of eptinezumab to the changes observed on patient-reported outcomes, though it has been shown that the preventive effect of eptinezumab can be observed as early as the day after infusion [28]. These analyses did not exclude patients who did not experience a new migraine during the 4-week treatment period, which occurred more frequently in the eptinezumab group than in the placebo group [16]. The effects of not experiencing another migraine attack in the study may over- or underestimate patient-reported evaluation of acute treatment optimization. In addition, the impact of specific items of the mTOQ-4 and HIT-6 were not explored; thus, it is unknown which factors of migraine impact were most affected by reduced acute treatment optimization. Lastly, the use of mTOQ-4 as the primary measure is somewhat limited in those patients who were optimized at baseline due to difficulty in assessing change from baseline in mTOQ-4 score.

## Conclusions

In this post hoc analysis, eptinezumab use was associated with greater improvements in response to acute treatment use after administration of eptinezumab during a migraine attack. Regardless of baseline treatment optimization, headache-related life impact also improved compared to placebo, suggesting that eptinezumab may work synergistically with acute medications and other treatments. These data highlight the potential benefit of eptinezumab in optimizing acute treatment use during breakthrough attacks and suggest that eptinezumab may reduce the overall impact and severity of migraine and potentially lower the risk of migraine chronification. Furthermore, the more pronounced results in poorly optimized patients indicate that those experiencing suboptimal acute medication effectiveness benefit the most from preventive treatment with eptinezumab.

## Data Availability

In accordance with EFPIA’s and PhRMA’s “Principles for Responsible Clinical Trial Data Sharing” guidelines, Lundbeck is committed to responsible sharing of clinical trial data in a manner that is consistent with safeguarding the privacy of patients, respecting the integrity of national regulatory systems, and protecting the intellectual property of the sponsor. The protection of intellectual property ensures continued research and innovation in the pharmaceutical industry. Deidentified data are available to those whose request has been reviewed and approved through an application submitted to https://www.lundbeck.com/global/our-science/clinical-data-sharing.

## Abbreviations

CGRP: Calcitonin gene-related peptide
HIT-6: 6-Item Headache Impact Test
ICHD-3: International Classification of Headache Disorders, 3^rd^ edition
MOH: Medication-overuse headache; mTOQ-4, 4-item Migraine Treatment Optimization Questionnaire
mTOQ-6: 6-Item Migraine Treatment Optimization Questionnaire
